# Enhanced Versatility of Table‐Top X‐Rays from Van der Waals Structures

**DOI:** 10.1002/advs.202105401

**Published:** 2022-03-31

**Authors:** Sunchao Huang, Ruihuan Duan, Nikhil Pramanik, Chris Boothroyd, Zheng Liu, Liang Jie Wong

**Affiliations:** ^1^ School of Electrical and Electronic Engineering Nanyang Technological University 50 Nanyang Avenue Singapore 639798 Singapore; ^2^ CINTRA CNRS/NTU/THALES UMI 3288 Research Techno Plaza Nanyang Technological University 50 Nanyang Avenue Singapore 637371 Singapore; ^3^ School of Materials Science and Engineering Nanyang Technological University 50 Nanyang Avenue Singapore 639798 Singapore; ^4^ Facility for Analysis Characterisation Testing, and Simulation (FACTS) Nanyang Technological University 50 Nanyang Avenue Singapore 639798 Singapore

**Keywords:** free electron radiation, nanomaterials, photonics, van der Waals materials, X‐rays

## Abstract

Van der Waals (vdW) materials have attracted much interest for their myriad unique electronic, mechanical, and thermal properties. In particular, they are promising candidates for monochromatic, table‐top X‐ray sources. This work reveals that the versatility of the table‐top vdW X‐ray source goes beyond what has been demonstrated so far. By introducing a tilt angle between the vdW structure and the incident electron beam, it is theoretically and experimentally shown that the accessible photon energy range is more than doubled. This allows for greater versatility in real‐time tuning of the vdW X‐ray source. Furthermore, this work shows that the accessible photon energy range is maximized by simultaneously controlling both the electron energy and the vdW structure tilt. These results will pave the way for highly tunable, compact X‐ray sources, with potential applications including hyperspectral X‐ray fluoroscopy and X‐ray quantum optics.

## Graphical abstract

Unprecedented versatility in van der Waals (vdW) materials as a platform for compact free electron‐driven X‐ray generation is demonstrated. Including the vdW material tilt angle as a controllable degree of freedom broadens the accessible X‐ray photon energy range by over 100%. This allows the tunable photon output to span the extreme ultraviolet and X‐ray regimes, using table‐top electron sources.

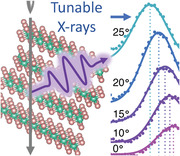

## 1 Introduction

Van der Waals (vdW) materials are a distinctive family of materials consisting of 2D sheets of atoms, either in single‐layer form (e.g., graphene), or in multilayer form, held together by vdW forces (e.g., graphite). Members of this family exhibit unique properties that can include linear energy‐momentum dispersion,^[^ ^1^ ^]^ giant intrinsic charge mobility,^[^ ^2^, ^3^ ^]^ extreme electromagnetic confinement,^[^ ^4^, ^5^ ^]^ quantum Hall effects,^[^ ^6^, ^7^, ^8^ ^]^ van Hove singularities,^[^ ^9^ ^]^ in‐plane and tunneling pressure sensors,^[^ ^10^ ^]^ gate‐tunable plasmons,^[^ ^11^ ^]^ tunable photon polaritons,^[^ ^12^ ^]^ and superconductivity.^[^ ^13^ ^]^ Among its many exciting prospects, vdW materials are promising platforms for nanomaterial‐based X‐ray sources. For example, the nanoscale electromagnetic confinement achievable in 2D vdW materials like graphene makes them promising platforms for compact, free electron‐driven sources of high‐brightness X‐rays.^[^ ^14^, ^15^, ^16^, ^17^ ^]^ Recently, the generation of tunable X‐rays from free electron‐driven vdW materials was theoretically predicted and experimentally demonstrated.^[^ ^18^ ^]^ The output X‐ray peaks can be tuned by controlling the electron kinetic energy and the atomic composition of the vdW material. X‐ray generation via electron‐crystal interaction also exists in conventional crystalline materials.^[^ ^19^, ^20^, ^21^, ^22^, ^23^, ^24^ ^]^ However, vdW materials and heterostructures^[^ ^25^, ^26^, ^27^, ^28^, ^29^, ^30^, ^31^ ^]^ are attractive platforms due to the large variety of compound combinations that provide control over the exact lattice constants determining the radiation spectrum. Besides, vdW materials have no dangling bonds or reconstruction at the surface,^[^ ^32^, ^33^ ^]^ and high in‐plane thermal conductivities,^[^ ^34^ ^]^ making them a compelling basis for compact, versatile, high‐quality X‐ray sources.^[^ ^18^, ^35^ ^]^

**Figure 1:**
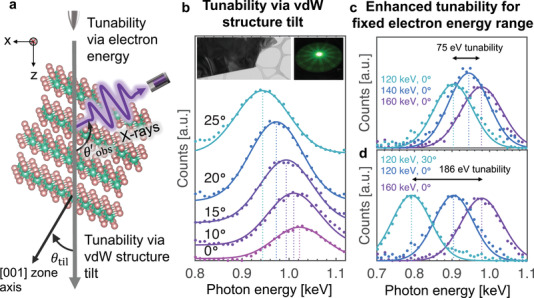
Enhanced tunability of X‐ray emission from van der Waals (vdW) materials by simultaneously varying the vdW structure tilt angle and the electron energy. a) An incident electron beam scatters off the periodic lattice of a vdW material, generating X‐rays via parametric X‐ray radiation and coherent bremsstrahlung. b) The spectra generated by a 200 keV electron beam impinging on a WSe_2_ single crystal (left top insert shows its transmission electron microscopesTEM image) at various tilt angles *θ*_til_, defined as the angle between the incident electron beam and the [001] zone axis. The angle is calibrated to within 0.5° of accuracy based on Kikuchi lines (right top insert). c,d) Enhanced tunability is achieved by simultaneously varying both the electron energy and the vdW structure tilt angle. The accessible X‐ray radiation photon energy range is more than doubled from 75 to 186 eV under the combined tuning scheme. In all panels, experimental results are represented by filled circles, and theoretical predictions by solid curves. Vertical dotted lines indicate the peak photon energy predicted by Equation (2). The estimated standard error for the photon count number is 1%, while that for the measured photon energy is 2.5 eV.

Here, we show that the versatility of the vdW‐based free electron‐driven X‐ray source can be significantly enhanced by combining the aforementioned tuning mechanisms—by electron energy and atomic composition—with a third mechanism: by varying the tilt angle of the vdW structure, denoted *θ*_til_ in **Figure** **1**a. Specifically, we theoretically predict and experimentally demonstrate that the range of accessible photon energies increases by over 100% when we simultaneously vary both the electron energy and the vdW structure tilt angle. This tilt angle is readily controlled by mechanically rotating the vdW structure with respect to the electron beam. In the process, we present a relativistic theory of free electron radiation in crystalline materials that accounts for all orders of emission processes within the same framework. We also demonstrate photon energy tuning via vdW structure tilt alone, showing that a wide range of photon energies can be accessed by varying the structure tilt angle at one fixed electron energy. Tuning the photon energy via the vdW structure tilt angle alone also has the advantage of being a simple mechanical maneuver that does not require re‐stabilizing and realigning the electron beam, as is the case when the electron energy is varied. The reason for re‐stabilization and re‐alignment is because we need a relatively collimated electron beam for the X‐ray generation mechanisms we study. Our results should pave the way for greater versatility in compact X‐ray sources based on vdW materials.

## 2 Results

Figure 1a illustrates the vdW‐based free electron‐driven X‐ray generation process. The passage of a free electron through a multilayered vdW structure modulates the bound electrons of the material's atoms, creating polarization currents that emit radiation via parametric X‐ray radiation (PXR). PXR can also be understood as the diffraction of the incident electron's Coulomb field off the periodic arrangement of atoms; in this respect, it is simply an atomic scale version of the Smith–Purcell radiation process.^[^ ^36^, ^37^, ^38^ ^]^ At the same time, the free electron itself is modulated by the periodic potential of the atomic lattice, resulting in photon emission via coherent bremsstrahlung (CB). In particular, CB results from the interference of emitted radiation from multiple, periodically spaced Bremsstrahlung events. These two types of X‐ray radiation (PXR and CB) share the same energy peak at a given detection angle, and are collectively termed parametric coherent bremsstrahlung (PCB).^[^ ^18^ ^]^ Due to the relatively low electron energies considered here (on the order of 100 keV; as opposed to MeV‐ or GeV‐scale kinetic energies), channeling radiation^[^ ^39^ ^]^ occurs in the visible–ultraviolet regime and does not contribute to the X‐ray output. In Figure 1a, the angle between the incident electron (along the *z*‐direction) and the [001] zone axis is denoted *θ*_til_, and is henceforth referred to as the vdW structure tilt angle.

We perform the experiments in transmission electron microscopes (TEM), measuring the emitted X‐rays using energy dispersive X‐ray spectroscopy (EDS) detectors, as detailed in Section 4. We obtain the average radiation intensity per electron of a large, incoherent electron beam as

(1) $$\begin{array}{c} \langle \frac{d^{2}N}{d\omega d\Omega }\rangle \approx \frac{1}{N_{e}}\frac{\alpha \omega }{4\pi^{2}c^{2}}\sum_{i=1}^{N_{e}}{\left|\int_{0}^{t_{L}}v_{i}\left(t\right)\cdot E_{ks}\left(r_{i},\omega \right)e^{-i\omega t}dt\right|}^{2} \end{array}$$

where *N* is the number of emitted photons, *ω* is the angular frequency of the emitted photon, Ω is the solid angle, *N*_e_ is the number of incident electrons, α is the fine‐structure constant, *c* is the speed of light in free space, *t*_*L*_ is the interaction time of the electron with the crystal, $v_{i}\left(t\right)$ is the velocity of electron, obtained via the relativistic Newton–Lorentz equation, $E_{ks}\left(r_{i},\omega \right)$ is an eigenmode of the crystal, k is the wave vector of the radiation field, *s* is the index of the polarization, and $r_{i}$ is the trajectory of the electron. Our derivation of Equation (1) is based on the scattering theory of Baryshevsky et al.,^[^ ^19^, ^40^, ^41^ ^]^ but importantly goes beyond it by: a) including relativistic corrections for the incident electron; b) summing over all the radiation arising from the various reciprocal lattice vectors g; and c) averaging over the initial positions of the electron on the crystal surface. Although we focus on electrons in this study, our theory is valid for any charged particle when the corresponding values for charge and rest mass are used. Our approach has advantages over approaches that consider PXR and CB using separate theoretical frameworks,^[^ ^18^ ^]^ as we are able to capture the effects of interference between PXR and CB processes, as well as the presence of higher‐order processes beyond PXR and CB. For details of the derivation, see Section S1, Supporting Information. We obtain the peak photon energy of the output X‐rays from the result of Equation (1) as

(2) $$\begin{array}{c} E=\;\hbar c\;\frac{\beta_{0}\hat{z}\cdot \left(\hat{U}g_{0}\right)}{1-\beta_{0}\cos\theta_{\mathrm{obs}}^{\prime }} \end{array}$$

where ℏ is the reduced Planck constant, *β*_0_ = *v*_0_/*c*, *v*_0_ being the initial speed of the incident electron, $\hat{z}\cdot \left(\hat{U}g_{0}\right)=\left(-\sin\phi_{\mathrm{til}}\cos\theta_{\mathrm{til}}\right)\;g_{0x}+\left(\sin\phi_{\mathrm{til}}\sin\theta_{\mathrm{til}}\right)\;g_{0y}+\left(\cos\theta_{\mathrm{til}}\right)\;g_{0z}$, where $\hat{U}$ is the unitary matrix and $g_{0}$ is the reciprocal lattice vector in the unrotated frame, that is, when *θ*_til_ = *ϕ*_til_ = 0°, *ϕ*_til_ is the rotation angle of the crystal with respect to the *z*‐axis and $\theta_{\mathrm{obs}}^{\prime }$ is the effective angle between the electron beam and the observation direction as shown in Figure 1a. Taking the X‐ray peak broadening due to electron beam divergence, the detector energy resolution, and the shadowing effect into account, we obtain the following expression for the measured bandwidth of the PCB peaks (see Section S1, Supporting Information for details):

(3) $$\begin{array}{ccc} \Delta E_{\mathrm{tot}} & \approx & [{\left(5.6\frac{\hbar v_{0}}{L}\right)}^{2}+{\left(\frac{\hbar c\beta_{0}\hat{z}\cdot \left(\partial \hat{U}g_{0}/\partial \theta_{\mathrm{til}}\right)}{1-\beta_{0}\cos\theta_{\mathrm{obs}}^{\prime }}\Delta \theta_{e}\right)}^{2}+R^{2}\\ & & {+E^{2}{\left(\frac{\beta_{0}\sin\theta_{\mathrm{obs}}^{\prime }}{1-\beta_{0}\cos\theta_{\mathrm{obs}}^{\prime }}\Delta \theta_{\mathrm{obs}}^{\prime }\right)}^{2}]}^{1/2} \end{array}$$

where *L* is the interaction length and *R* is the energy resolution of the EDS detector. In determining the actual observation angle $\theta_{\mathrm{obs}}^{\prime }$ and its angular spread $\Delta \theta_{\mathrm{obs}}^{\prime }$ in Equation (3), we take into account the shadowing effect, which causes the effective observation angle to increase (the effective observation angular spread to decrease) by a few degrees from its default value *θ*_obs_ (Δ*θ*_obs_). This deviation is due to the edge of the sample holder partly blocking the output X‐rays on their way toward the EDS detector^[^ ^42^ ^]^ (see Section S3, Supporting Information, for more details). The first term in Equation (3) corresponds to the intrinsic bandwidth of the PCB X‐ray peak^[^ ^40^ ^]^ obtained from Equation (1), which is on the order of 1 eV in our case. The second term corresponds to effects of electron beam divergence. In our experiments the beam divergence Δ*θ*_e_ ≈ 1 mrad. The third term accounts for the energy resolution of the EDS detector. The final term accounts for the finite range of observation directions admitted by the angular aperture of the EDS detector. Figure 1 shows good agreement between the experimental measurements (filled circles) with the predictions of our theory (solid lines).

Figure 1b shows the PCB spectrum when a 200 keV electron beam is incident on a WSe_2_ single crystal. The X‐ray photon energy is tuned from 1026 to 946 eV when the tilt angle of the WSe_2_ single crystal is varied from *θ*_til_ = 0° to *θ*_til_ = 25°, where we determine *θ*_til_ to an accuracy better than 0.5° in the experiments by using Kikuchi lines.^[^ ^43^, ^44^ ^]^ Kikuchi lines are produced by Bragg reflections of inelastically scattered electrons in crystals, which provide an effective way to accurately measure crystal orientation. For hexagonal close packed crystals such as WSe_2_ and MoS_2_, the overlap between the [001] zone‐axis and the incident electron beam results in bright lines (Kikuchi lines) that are distributed evenly around a central point (right insert in Figure 1b).^[^ ^45^ ^]^ Tuning via vdW structure tilt would be helpful in scenarios where other tuning mechanisms are not as readily available: for instance, tuning via the electron energy typically requires readjustment of the accelerating voltage and realigning of the electron beam; whereas tuning via atomic composition requires the growth of a completely new material. A TEM image of our WSe_2_ sample is shown in the left insert of Figure 1b. In Figure 1c,d, we consider an electron energy range of 120–160 keV. Figure 1c shows that the achievable output photon energy range is 75 eV when *θ*_til_ = 0° and only the electron energy is allowed to vary. In Figure 1d, this range increases by over 100% to 186 eV when we allow the electron energy and the vdW structure tilt angle to simultaneously vary.

**Figure 2:**
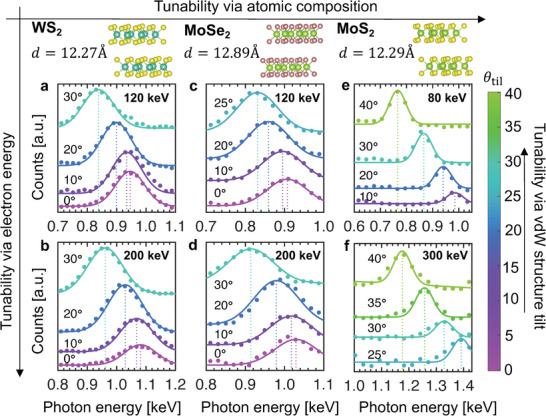
3D tunability of free electron radiation in van der Waals (vdW) materials. Tunability via vdW structure tilt, electron energy, and atomic composition. a–f) Together illustrate our paradigm of a highly versatile, compact X‐ray source in which the photon energy can be tuned over a wide range by varying the structure tilt, the electron energy and the atomic composition. X‐ray spectra from WS_2_ (a,b), MoSe_2_ (c,d) and MoS_2_ (e,f) are tailored by varying electron energy (labeled at top right corner of each panel) and structure tilt angle *θ*_til_ (labels individual curves within each panel). Filled circles represent experimental measurements and solid curves correspond to theoretical predictions. Vertical dotted lines indicate the peak photon energy predicted by Equation (2). Comparing (b) and (d), WS_2_ with a smaller interlayer spacing generates harder X‐rays compared to MoSe_2_ under the same conditions, showing the tunability of vdW X‐rays via atomic composition. In (a–d), *θ*_obs_ ≈ 112.5° and Δ*θ*_obs_ ≈ 12°. In (e,f), *θ*_obs_ ≈ 91.5° and Δ*θ*_obs_ ≈ 1°. In all cases, the intrinsic bandwidth is about a few eV, but is broadened by the energy resolution of the respective EDS detectors. The estimated standard error for the photon count number is 1%, while that for the measured photon energy is 2.5 eV.

Dichalcogenide vdW materials like WSe_2_, WS_2_, and MoS_2_ crystallize in a layered structure with slightly differing interlayer distances,^[^ ^46^ ^]^ which offer opportunities to tune the output X‐ray photon energy via atomic composition. ^[^ ^18^ ^]^ Combined with tunability via the vdW structure tilt and the electron energy, this makes vdW materials a versatile platform for compact X‐ray generation. **Figure** **2** shows 3D tunability of the vdW X‐ray radiation: tunability via the electron energy, tunability via the atomic composition, and tunability via the vdW structure tilt. Tunability via the atomic composition allows the pre‐customization of a PCB X‐ray source by choosing the constituents of the vdW structure. The many compound combinations possible in vdW materials provide precise control over the lattice constants that determine the radiation spectrum. On the other hand, tunability via the electron energy and via the vdW structure tilt provides dynamic tunability: the electron energy can be adjusted by changing the accelerator voltage of the electron source, and the vdW structure tilt angle can be adjusted by mechanical rotation. It should be noted that the intrinsic bandwidth of the PCB peaks is also very narrow, being in the order of 1 eV in our regime of study. The measured bandwidth is significantly broadened by the large energy resolutions and observation angle spreads of the respective EDS detectors. It should be noted that our demonstrated energy tunability (≈200 eV) greatly exceeds the intrinsic bandwidth of our X‐ray source (≈1 eV). Furthermore, as we also show below, a much larger range of X‐ray photon energy tunability (>10 keV and more) can be achieved at observation angles and electrons energies beyond what we can access in our electron microscopes—but still on a table‐top scale. The linewidth dependence of the X‐ray peaks is as described by Equation (3). It should be noted that in our experiments, the dominant contribution of the EDS detector's energy resolution and angular range (see Section 4) eclipses the dependence of the X‐ray peak linewidth on other factors such as electron energy and vdW tilt angle. A potential way to directly measure the narrow linewidth of the PCB peaks is by using Bragg's law‐based techniques such as wavelength‐dispersive X‐ray spectroscopy, whose energy resolution can be on the order of 1 eV—instead of the EDS measurements we perform here.

**Figure 3:**
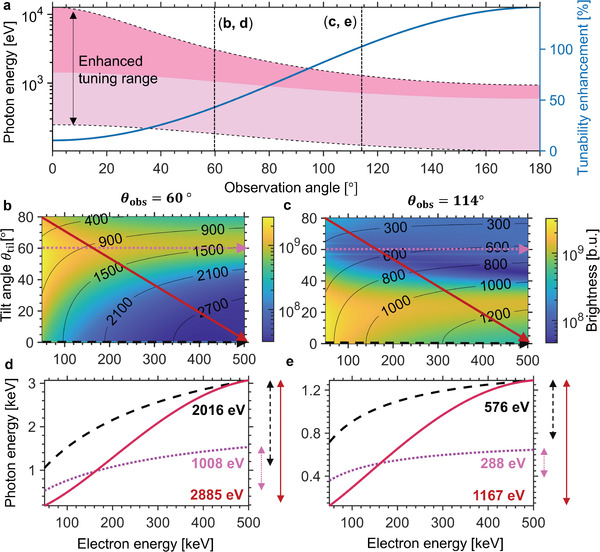
Enhanced tunability for various observation angles in WS_2_. a) The entire shaded region between the two dashed lines corresponds to the accessible photon energy range when vdW structure tilt and electron energy are simultaneously varied, whereas the darker pink shaded portion corresponds to that when only the electron energy is varied. The blue curve represents the percentage enhancement in the photon energy range in the former scheme. b,c) Brightness (Equation (1)) as a function of electron energy for *θ*_til_ from 0° to 80° at different *θ*_obs_, where “brightness units”, (b.u.) stands for photons s^−1^ mm^−2^ mrad^−2^ per 0.1% BW. The photon energy of X‐rays (in eV) is indicated by the black contour lines (Equation (2)). d,e) Accessible photon energy range by tuning along the arrows in colormaps (b,c) respectively. Our enhanced tunability especially favors emission at obtuse detector angles and in the soft X‐ray range. In this figure, *ϕ*_til_ = 0° and *L* = 100 nm.

**Figure** **3**a depicts the X‐ray photon energy range accessible with the vdW‐based X‐ray source. If only the electron energy is allowed to vary, only photon energies in the dark pink shaded region can be accessed. This region becomes increasingly narrow at larger observation angles, which favor softer X‐rays that could be beneficial for biological imaging. On the other hand, if the electron energy is varied together with vdW structure tilt angle, we see that the accessible range of output X‐ray photon energies expands to the entire pink‐shaded region, bounded by the pair of dashed lines. The solid blue line in Figure 3a reflects the percentage enhancement in accessible photon energy range by combining control over both electron energy and vdW structure tilts. This percentage enhancement is well in excess of 100% at larger detector angles. Here, we have considered an electron source that can be tuned from 50 to 500 keV (which covers the electron energy range of most TEMs). Figures 3b,d and 3c,e focus on the specific cases where *θ*_obs_ = 60° and *θ*_obs_ = 114°, respectively. In both cases (as in all other cases used in Figure 3a), the tuning scheme via electron energy and vdW structure tilt runs diagonally across the range of vdW structure tilt and electron energies considered (red lines in Figure 3b,c). The resulting photon energy peaks are shown in Figure 3d,e respectively, and contrasted against cases where only the electron energy is allowed to vary (horizontal lines in Figure 3b,c). At the same time, the colormaps in Figure 3b,c shows the brightness of the output X‐ray photons, as calculated from Equation (1). We see that the brightness can vary significantly across the entire tuning range. For any specific output X‐ray photon energy, it is possible to maximize the X‐ray brightness with the freedom to vary both electron energy and vdW structure tilt angle. Simultaneously controlling both electron energy and vdW structure tilt thus allows us to optimize the accessible photon energy range as well as the intensity of the vdW X‐ray source.

For relativistic electrons (1–10 MeV), tuning by varying the electron energy becomes challenging at observation angles beyond 20°, as discussed in Section S2, Supporting Information. The only feasible way to tune the photon energy in real‐time for relativistic electrons is via the vdW structure tilt angle. Specifically, tuning via the vdW structure tilt angle allows us to enhance the emitted photon energy range by 1873% and 654% for *θ*_obs_ = 114° and *θ*_obs_ = 60° respectively, compared to tuning by varying the electron energy.

## 3 Discussion

The vdW X‐ray generation scheme we study is highly complementary to other existing methods of X‐ray generation. The vdW X‐ray source is dynamically tunable in frequency, unlike traditional X‐ray tubes whose output peaks are fixed at the characteristic frequencies of the anode material.^[^ ^47^ ^]^ Furthermore, it requires neither highly relativistic electrons nor high intensity lasers, as in undulator‐based X‐ray sources^[^ ^48^ ^]^ and high‐harmonic generation.^[^ ^15^ ^]^ Our results should pave the way for the realization of dynamically tunable, compact X‐ray sources, which have a wide range of potential applications in imaging and inspection,^[^ ^49^, ^50^, ^51^ ^]^ including X‐ray hyperspectral imaging and X‐ray quantum optics.^[^ ^52^, ^53^, ^54^, ^55^ ^]^ In particular, applications for narrowband X‐rays already include X‐ray diffraction and near‐edge X‐ray absorption fine structure measurements. Our source has the potential to serve these applications, but with the added benefits of dynamic photon energy tunability and potentially higher brightness. The study of shaping of incident free electrons^[^ ^56^, ^57^, ^58^, ^59^, ^60^, ^61^, ^62^, ^63^, ^64^, ^65^, ^66^, ^67^ ^]^ —on the level of either the macroscopic bunch structure or the individual electron wavefunction—is a subject of active investigation that could lead to greater control and enhancement of the output radiation.

In our experiments, we measured 7.9 × 10^4^ PCB photons over a duration of 1000 s (live time) from WS_2_ at *θ*_til_ = 30°, shown in Figure 2b. This yields a flux of 79 photons s^−1^, which is in excellent agreement with our theory for a current of 0.34 nA. The relatively low electron current was used to avoid pileup effects during the measurement of X‐rays by the EDS detector, whose dead time was kept below 30%. This scales to a brightness of ≈1 × 10^9^ photons s^−1^ mm^−2^ mrad^−2^ per 0.1% BW when an electron beam of 1 nA current and 1 nm spot size is employed, which is consistent with the results reported in ref. [18]. This brightness also compares favorably with that of high harmonic generation (10^5^ − 10^12^ photons s^−1^ mm^−2^ mrad^−2^ per 0.1% BW) in the water‐window.^[^ ^68^, ^69^ ^]^ The angular flux density from our unoptimized source is about ≈ 4 × 10^6^ *I*(A) (photons s^−1^ mrad^−2^ per 0.1% BW), which already comes close to that of conventional X‐ray tubes ≈ 10^7^ − 10^8^ *I*(A) (photons s^−1^ mrad^−2^ per 0.1% BW),^[^ ^70^ ^]^ where *I*(A) refers to the current in Amperes. The thickness of our sample is about 100 nm, which corresponds to a few hundred atomic layers. The radiation intensity can be enhanced by increasing the sample thickness, resulting in larger interaction length *L*. It should be noted that the peak brightness is directly proportional to the square of the interaction length (i.e., *L* ^2^). Larger interaction lengths, however, come at the cost of broadened X‐ray peaks due to deterioration in the quality of the electron beam as it travels through more of the material structure. The deterioration of the electron beam is in turn due to increased scattering events, which cause electrons to lose energy and/or be deflected from their original direction of travel. The X‐ray peak brightness is also directly proportional to electron current. As in X‐ray tubes, a larger electron current will generate more heat and increase the possibility of thermal damage. In this regard, vdW materials like graphite have an advantage over conventional materials (e.g., tungsten, commonly used as the anode in X‐ray tubes) due to the former's superior thermal conductivity and melting point. Based on our experimental results, it should be noted that just increasing the current to 1 mA – which is typical for X‐ray tubes – already results in an X‐ray photon flux in excess of 10^8^ photons per s, sufficient for X‐imaging applications. Innovative methods to increase the interaction length include having the electrons travel near the edge of vdW materials in a Smith–Purcell‐like configuration that has been termed edge PXR.^[^ ^35^ ^]^ This allows the electron's Coulomb fields to scatter off the crystal lattice while minimizing collisions of the electrons themselves with the material. In our experiments, the measured PCB peak intensity is about 100 times larger than that of incoherent bremsstrahlung. Since the intensity of incoherent bremsstrahlung scales as ∝*L*, we expect this ratio to increase when longer interaction distances are considered.

In conclusion, we have shown that the versatility of the vdW‐based free electron X‐ray source can be significantly enhanced with the introduction of a new control parameter: the vdW structure tilt angle, which can be varied in real‐time by mechanically rotating the vdW target with respect to the electron beam. Specifically, we show that the range of accessible photon energies increases by over 100% when we simultaneously vary both the electron energy and the vdW tilt angle. At the same time, we present a relativistic theory of PCB that not only accounts for both PXR and CB in the same framework, but also includes arbitrarily higher‐order free electron radiation processes. This, combined with the ability to tailor the vdW‐based X‐rays via atomic composition, makes van der Waals materials a promising platform for highly versatile, tunable X‐ray sources. Our results also show that a wide range of photon energies can be accessed just by varying the vdW tilt angle alone, even with a fixed electron energy and atomic composition. Although our study focuses on moderate electron energies (0.05–10 MeV), our method of enhancing the photon energy range by combining control over electron energy and tilt angle applies to other ranges of electron energies, and also other crystalline material systems beyond vdW materials. Our results should pave the way to realizing compact sources of high quality X‐rays for applications including hyperspectral X‐ray fluoroscopy and X‐ray quantum optics.

## 4 Experimental Section

### Sample Preparation

2D bulk MX_2_ (M= Mo, W; X = S, Se) single crystals were synthesized by the normal chemical vapor transport method. The stoichiometric ratio of high purity M and X with a bit of iodide as transport agent were loaded in a silica tube, which was sealed in a high vacuum environment. The sealed silica tube was loaded in a two‐zone furnace, whose growth zone was heated to 850 °C and reaction zone was heated to 950 °C within 24 h, and held for 10 days. Finally, bulk MX_2_ single crystals were collected in the growth zone. The few‐layer MX_2_ nanoflakes were exfoliated mechanically onto silicon substrates (covered with a 285 nm SiO_2_ film), and transferred to Au grids (for TEM measurement) with the aid of the wet‐transfer method. 

### X‐Ray Measurements

The vdW‐based X‐ray emission measurements were conducted in TEM. A highly collimated electron beam was sent toward the vdW material in the sample holder, which could be tilted. The emitted X‐ray spectra were measured using a silicon drift EDS detector. The EDS detector was calibrated by the authors to enable measurement of X‐ray peak energies with an accuracy of ±2.5 eV (see Section S4, Supporting Information, for details). The experiments shown in Figures 1 and 2a–d were conducted in a JEOL 2010HR TEM, which used 120–200 keV electrons. In the photon energy range of interest (0.7–1.4 keV), the energy resolution was *R* ≈ 97 eV for the EDS detector in the JEOL 2010HR TEM. The detector's observation angle and observation angle range were *θ*_obs_ ≈ 112.5° and Δ*θ*_obs_ ≈ 12°, respectively. The experiments in Figure 2e,f were performed in a JEM‐ARM300F TEM, which used 80 and 300 keV electrons. In the photon energy range of interest, energy resolution *R* ≈ 75 eV for the EDS detector in a JEM‐ARM300F TEM. In both TEMs, the sample holder was made of beryllium, and could be rotated about the *x*‐ and *y*‐axes (the *x*–*y* plane being that which lies parallel to the surface of the sample holder), that allowed to determine *θ*_til_ to an accuracy better than 0.5° with the help of Kikuchi lines. In all measurements, increasing *θ*_til_ further tilted the sample toward the EDS detector. The range of *θ*_til_ was ±30° and ±40° for the JEOL 2010 HR TEM and the JEM‐ARM300F TEM, respectively. In the measurements, the electron beam divergence was about 1 mrad, the spot size on the sample was about 10 nm, and the beam current was about 0.3 nA.

### Theory and Simulations

See Section S1, Supporting Information, for details.

### Statistical Analysis

The background radiation of the measured spectra was subtracted using NIST DTSA‐II.^[^ ^71^, ^72^ ^]^ All statistical tests in this study were performed using MATLAB. The estimated standard error for the X‐ray count number was 1%, while that for the measured photon energy was 2.5 eV, which were also provided in the corresponding figure legends.

## Conflict of Interest

The authors declare no conflict of interest.

## Author Contributions

S.H. led the project and analyzed the data. S.H. designed and performed the X‐ray measurements with the help and advice of C.B., who also performed some of the measurements. R.D. prepared all the samples with the help of Z.L. N.P. developed the theory and performed the simulations with the help of S.H. and L.J.W. S.H., N.P., and L.J.W. wrote the paper, with inputs from all other authors. L.J.W. conceived the idea and supervised the project.

## Supporting information

Supporting Information

## Data Availability

The data that support the findings of this study are available from the corresponding author upon reasonable request.
