# Supplementary material for: Enhanced Versatility of Table‐Top X‐Rays from Van der Waals Structures
Source: Adv Sci (Weinh). 2022 Mar 31;9(16):2105401. doi: 10.1002/advs.202105401 (PMC9165495; doi:10.1002/advs.202105401)
Supplement: Supplementary file 1 — Supporting Information [file ADVS-9-2105401-s001.pdf]

ADVANCED SCIENCE
Open Access

Supporting Information

for *Adv. Sci.*, DOI 10.1002/advs.202105401

Enhanced Versatility of Table-Top X-Rays from Van der Waals Structures

*Sunchao Huang, Ruihuan Duan, Nikhil Pramanik, Chris Boothroyd, Zheng Liu and Liang Jie Wong**

Supplementary Information

Enhanced versatility of table-top X-rays from van der Waals structures

Sunchao Huang, Ruihuan Duan, Nikhil Pramanik, Chris Boothroyd, Zheng Liu and Liang Jie Wong*

*Email: liangjie.wong@ntu.edu.sg

1 Theory of free electron radiation in crystals

In this section, we present the theory for light emission from a uniformly distributed electron beam incident upon a crystalline structure. As these derivations are obtained *ab initio* from Maxwell's equations, our results are valid within the realm of classical relativistic electrodynamics, as long as electron recoil and quantization of electron momenta (as a result of traveling in the periodic potential of the crystalline lattice) is insignificant, which is true in our regime of interest. The single-electron treatment is based on the scattering theory of Baryshevsky et al. [1], but with the generalization of the formulas to include relativistic electrons. Furthermore, we take into account all the (hkl) planes which make considerable contributions to the intensity of photons emitted with frequency ω at a direction $\hat{\mathbf{n}}$. The radiation spectrum per electron is then obtained by averaging over the radiation spectra of uniformly distributed electrons spanning the crystal surface, where we increase the number of impinging electrons until the convergence is achieved. Although we focus on electrons in our theory, our results are valid for any charged particle when the corresponding values for charge and rest mass are used.

1.1 Statistically averaged photon emission spectrum

In this subsection, we show the average radiation intensity emitted by an electron passing through a crystalline material. Using the Pointing vector, in the far-field regime, the radiation intensity is given by[2]

$$\frac{\mathrm{d}^2 N}{\mathrm{d}\omega \mathrm{d}\Omega} = \frac{4\pi c \epsilon_0}{\hbar\omega} \mathcal{R}^2 |\boldsymbol{E}(\boldsymbol{r}, \omega)|^2, \tag{S1}$$

where N is the number of emitted photons, ω is the angular frequency of the emitted photon, Ω is the solid angle, c is the speed of light in free space, ϵ_0 is the vacuum permittivity, $\hbar$ is the reduced Planck's constant, $\mathcal{R}$ is the distance between the charge and the observation point, $\boldsymbol{r}$ refers to the position vector in space, $\boldsymbol{E}(\boldsymbol{r}, \omega)$ is the radiation electric field. We obtain $\boldsymbol{E}(\boldsymbol{r}, \omega)$ by solving the wave equation (derived from the Maxwell's equations in matter (non-magnetic linear media))[2]

$$\boldsymbol{\nabla} \times \boldsymbol{\nabla} \times \boldsymbol{E}(\boldsymbol{r}, \omega) - \omega^2 \mu_0 \boldsymbol{D}(\boldsymbol{r}, \omega) = \mathrm{i}\omega\mu_0 \boldsymbol{J}(\boldsymbol{r}, \omega), \tag{S2}$$

where μ_0 is the vacuum permeability, which is considered to be the permeability of the non-magnetic material, $\boldsymbol{D}(\boldsymbol{r}, \omega) = \epsilon(\boldsymbol{r}, \omega)\boldsymbol{E}(\boldsymbol{r}, \omega)$ is the local electric displacement, $\epsilon(\boldsymbol{r}, \omega)$ being the permittivity of the material, $\boldsymbol{J}(\boldsymbol{r}, \omega) = \frac{1}{2\pi} \int \mathrm{d}t \; q\boldsymbol{v}(t) \; \delta(\boldsymbol{r} - \boldsymbol{s}(t)) \exp(\mathrm{i}\omega t)$ is the free current density, where $\boldsymbol{v}(t)$ and $\boldsymbol{s}(t)$ are the velocity and position of the moving charge q. The non-homogeneous differential equation (S2) is solved using the Green's function method [3]. The component of the emitted radiation field is then given as

$$E_i(\boldsymbol{r}, \omega) = \mathrm{i}\omega\mu_0 \int_{-\infty}^{\infty} \mathrm{d}^3\boldsymbol{r}' \sum_j G_{ij}(\boldsymbol{r}, \boldsymbol{r}', \omega) J_j(\boldsymbol{r}', \omega), \tag{S3}$$

where the indices i,j = x, y, z, the position of the current source is denoted $\boldsymbol{r}'$. In the limit $\boldsymbol{r} >> \boldsymbol{r}'$, the element of the Green's tensor is given as [1]

$$G_{ij}(\boldsymbol{r},\boldsymbol{r}',\omega) \approx \frac{1}{4\pi}\frac{\exp(\mathrm{i}kr)}{r}\sum_{s=1,2}\hat{\boldsymbol{e}}_{s_i}(\boldsymbol{k})E^*_{\boldsymbol{k}s_j}(\boldsymbol{r}',\omega), \tag{S4}$$

where $k = \omega/c$, $\hat{\boldsymbol{e}}_{s_i}$ is the ith component of the polarisation vector, s is the index of polarisation and $E^*_{\boldsymbol{k}s_j}(\boldsymbol{r}',\omega)$ refers to the complex conjugate of the jth component of the crystal eigenmode $\boldsymbol{E}_{\boldsymbol{k}s}$, where $\boldsymbol{k}$ denotes wave vector. The crystal eigenmodes satisfy the homogeneous differential equation (the free current density in equation (S2) is set to 0)

$$\boldsymbol{\nabla}\times\boldsymbol{\nabla}\times\boldsymbol{E}_{\boldsymbol{k}s}(\boldsymbol{r},\omega) - \omega^2\mu_0\,\epsilon(\boldsymbol{r},\omega)\boldsymbol{E}_{\boldsymbol{k}s}(\boldsymbol{r},\omega) = 0, \tag{S5}$$

where the permittivity $\epsilon(\boldsymbol{r},\omega)$ can be expanded as $\epsilon(\boldsymbol{r},\omega) = \epsilon_0 + \epsilon'(\boldsymbol{r},\omega)$, where $\epsilon'(\boldsymbol{r},\omega) = \epsilon_0\chi(\boldsymbol{r},\omega)$. Here, $\chi(\boldsymbol{r},\omega) = \sum_{\boldsymbol{g}\neq 0}\chi_{\boldsymbol{g}}\exp(\mathrm{i}\boldsymbol{g}\cdot\boldsymbol{r})$ is the electric susceptibility [4] of the crystal, $\chi_{\boldsymbol{g}}$ is the Fourier component of electronic susceptibility and $\boldsymbol{g}$ is the reciprocal lattice vector. For X-ray photons, the vacuum permittivity $\epsilon_0 >> \epsilon'(\boldsymbol{r},\omega)$ because $\chi_{\boldsymbol{g}}$ is very small ($\sim 10^{-3}$) as seen in Fig. S1. The electric field eigenmodes is expanded in a perturbative manner as

$$\boldsymbol{E}_{\boldsymbol{k}s}(\boldsymbol{r},\omega) = \boldsymbol{E}_0(\boldsymbol{r},\omega) + \boldsymbol{E}'(\boldsymbol{r},\omega), \tag{S6}$$

where $\boldsymbol{E}_0(\boldsymbol{r},\omega)$ is the unperturbed field and $\boldsymbol{E}'(\boldsymbol{r},\omega)$ is the small perturbation. In the limit, where $\epsilon_0 >> \epsilon'(\boldsymbol{r},\omega)$ and $|\boldsymbol{E}_0(\boldsymbol{r},\omega)| >> |\boldsymbol{E}'(\boldsymbol{r},\omega)|$, the expression for the electric field eigenmodes in a crystalline material is given as [5]

$$\boldsymbol{E}_{\boldsymbol{k}s}(\boldsymbol{r},\omega) = \exp(\mathrm{i}\boldsymbol{k}\cdot\boldsymbol{r})\hat{\boldsymbol{e}}_s + \sum_{\boldsymbol{g}\neq 0}\boldsymbol{E}_{\boldsymbol{g}s}e^{\mathrm{i}(\boldsymbol{k}+\boldsymbol{g})\cdot\boldsymbol{r}}, \tag{S7}$$

$$\boldsymbol{E}_{\boldsymbol{g}s} = -\chi_{\boldsymbol{g}}\frac{\left[\boldsymbol{k}_{\boldsymbol{g}}(\boldsymbol{g}\cdot\hat{\boldsymbol{e}}_s) - \frac{\omega^2}{c^2}\hat{\boldsymbol{e}}_s\right]}{({k_{\boldsymbol{g}}}^2 - \frac{\omega^2}{c^2})}, \quad \boldsymbol{k}_{\boldsymbol{g}} = \boldsymbol{k}+\boldsymbol{g}, \tag{S8}$$

$$\chi_{\boldsymbol{g}} = -\frac{1}{4\pi\epsilon_0}\frac{4\pi e^2}{m_e\omega^2}\frac{S(\boldsymbol{g})}{V}\exp[-W(\boldsymbol{g})], \tag{S9}$$

where $\hat{\boldsymbol{e}}_s$ is the polarization vector, m_e is the mass of an electron, $S(\boldsymbol{g}) = \sum_i F_i(\boldsymbol{g}) \exp(i\boldsymbol{g}\cdot\boldsymbol{R}_i)$ is the structure factor, where the form factor and the coordinate of the ith atom in the unit cell are denoted $F_i(\boldsymbol{g})$ and $\boldsymbol{R}_i$ respectively, e is the charge of an electron, V is the volume of the unit cell and the term $\exp[-W(\boldsymbol{g})]$ is the Debye-Waller factor[6]. This factor accounts for the effect of thermal vibrations in the crystal, whose value is approximately equal to 1 in our regime of study.

By substituting equation (S3) in equation (S1), we obtain the radiation intensity per electron. Then, the average radiation intensity per electron is obtained by averaging over the radiation intensity of uniformly distributed electrons impinging at different locations on the crystal surface, given as equation (1) of the main text.

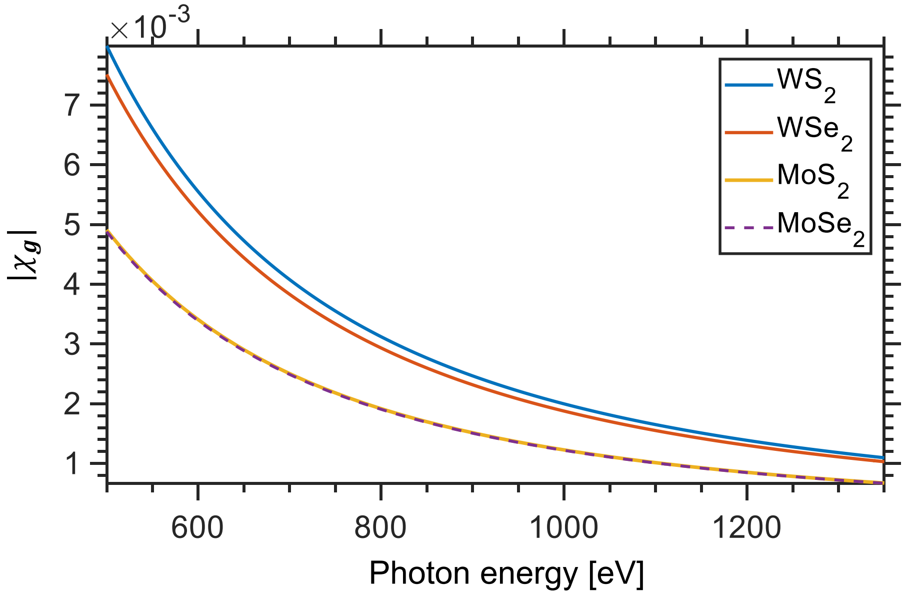

Figure S1: **Photon energy dependence of $\chi_{\boldsymbol{g}}$ for various van der Waals materials** (calculated from equation S9) [4]. In this plot, $\boldsymbol{g}$ is the reciprocal lattice vector corresponding to (002) planes. The lattice constant c for WS_2, WSe_2, MoS_2 and $MoSe_2$ are taken as 12.27Å, 12.98Å, 12.29Å and 12.89Å, respectively.

1.2 Dynamics of an electron in crystalline materials

In this subsection, we derive the trajectory and velocity of an electron passing through a crystalline material. The Newton-Lorentz equation of motion governs the motion of charged particles in the Coulomb field of periodically arranged atoms in a crystal, which is given as

$$\frac{\mathrm{d}\boldsymbol{p}}{\mathrm{d}t} = q\boldsymbol{\mathcal{E}} \tag{S10}$$

in the absence of magnetic fields, where $\boldsymbol{p} = \gamma m \boldsymbol{v}$ is the relativistic momentum, γ is the Lorentz factor, m is the mass, $\boldsymbol{v}$ is the velocity, q is the charge (an electron for our case), $\boldsymbol{\mathcal{E}} = -\nabla U(\boldsymbol{r})$ is the electric field experienced by the charge in the crystal. $U(\boldsymbol{r})$ is the potential due to the electron density and the atomic nucleus in the crystal lattice, given as[7]

$$U(\boldsymbol{r}) = \frac{1}{2V}\sum_{\boldsymbol{g}\neq 0} U_{\boldsymbol{g}} \exp(\mathrm{i}\boldsymbol{g}\cdot\boldsymbol{r}) + \text{c.c.}, \tag{S11}$$

$$U_{\boldsymbol{g}} = \frac{\mathrm{e}}{\epsilon_0}\sum_i \exp(\mathrm{i}\boldsymbol{g}\cdot\boldsymbol{R}_i)\frac{Z_i - F_i(\boldsymbol{g})}{g^2}\exp[-W(\boldsymbol{g})], \tag{S12}$$

where Z_i is the ith atom's nuclear charge, and c.c. denotes complex conjugate. We simplify equation (S10) to obtain the equation of motion for the x, y, z components as

$$\begin{aligned} \frac{\mathrm{d}v_\mathrm{x}}{\mathrm{d}t} &\approx \frac{q}{\gamma m}\,\mathcal{E}_\mathrm{x}, \\ \frac{\mathrm{d}v_\mathrm{y}}{\mathrm{d}t} &\approx \frac{q}{\gamma m}\,\mathcal{E}_\mathrm{y}, \\ \frac{\mathrm{d}v_\mathrm{z}}{\mathrm{d}t} &\approx \frac{q}{\gamma^3 m}\,\mathcal{E}_\mathrm{z}, \end{aligned} \tag{S13}$$

where we have assumed that a) the transverse velocity modulations of the electron are negligible such that $x \approx x_0$, $y \approx y_0$ and $\gamma \approx 1/\sqrt{1-(v_\mathrm{z}/c)^2}$, b) the longitudinal velocity modulations are negligible such that $z \approx z_0 + v_0 t$, v_0 being the initial velocity of the electron

which we consider to be along the z direction without loss of generality. Here, x, y and z refers to the position of the electron in Cartesian coordinates at time t, and the subscript "0" in these variables denotes its values at initial time.

The trajectory of the electron inside the crystal is given as

$$\boldsymbol{r} = \boldsymbol{r}_0 + v_0 t\ \hat{\mathbf{z}} + \delta_{\boldsymbol{r}}, \tag{S14}$$

where $\boldsymbol{r}_0 = x_0\hat{\mathbf{x}} + y_0\hat{\mathbf{y}} + z_0\hat{\mathbf{z}}$ is the initial position of the electron, and $\delta_{\boldsymbol{r}}$ is the fluctuation in the electron's position. In the limit $\delta_r << v_0 t$, which is true in our regime of interest, we analytically evaluated equation (S13) to obtain the electron's velocity components as

$$v_{\mathrm{x}} \approx \sum_{\boldsymbol{g}\neq 0} \frac{g_x}{\gamma}\zeta_g \left(\frac{e^{\mathrm{i}(\boldsymbol{g}\cdot v_0\hat{\mathbf{z}})t} e^{\mathrm{i}(\boldsymbol{g}\cdot\boldsymbol{r}_0)} + \mathrm{c.c.}}{2} \right), \tag{S15}$$

$$v_{\mathrm{y}} \approx \sum_{\boldsymbol{g}\neq 0} \frac{g_y}{\gamma}\zeta_g \left(\frac{e^{\mathrm{i}(\boldsymbol{g}\cdot v_0\hat{\mathbf{z}})t} e^{\mathrm{i}(\boldsymbol{g}\cdot\boldsymbol{r}_0)} + \mathrm{c.c.}}{2} \right), \tag{S16}$$

$$v_{\mathrm{z}} \approx \sum_{\boldsymbol{g}\neq 0} \frac{g_z}{\gamma^3}\zeta_g \left(\frac{e^{\mathrm{i}(\boldsymbol{g}\cdot v_0\hat{\mathbf{z}})t} e^{\mathrm{i}(\boldsymbol{g}\cdot\boldsymbol{r}_0)} + \mathrm{c.c.}}{2} \right) + v_0, \tag{S17}$$

where $\zeta_g = \frac{-qU_{\boldsymbol{g}}}{(\boldsymbol{g}\cdot\boldsymbol{v}_0)mV}$ and g_i is the ith component of $\boldsymbol{g}$. Then we obtain the fluctuations in position as

$$\delta_x \approx \sum_{\boldsymbol{g}\neq 0} \frac{g_x}{\gamma} \frac{\zeta_g}{(\boldsymbol{g}\cdot v_0\hat{\mathbf{z}})} \left(\frac{-\mathrm{i}e^{\mathrm{i}(\boldsymbol{g}\cdot v_0\hat{\mathbf{z}})t} e^{\mathrm{i}(\boldsymbol{g}\cdot\boldsymbol{r}_0)} + \mathrm{c.c.}}{2} \right), \tag{S18}$$

$$\delta_y \approx \sum_{\boldsymbol{g}\neq 0} \frac{g_y}{\gamma} \frac{\zeta_g}{(\boldsymbol{g}\cdot v_0\hat{\mathbf{z}})} \left(\frac{-\mathrm{i}e^{\mathrm{i}(\boldsymbol{g}\cdot v_0\hat{\mathbf{z}})t} e^{\mathrm{i}(\boldsymbol{g}\cdot\boldsymbol{r}_0)} + \mathrm{c.c.}}{2} \right), \tag{S19}$$

$$\delta_z \approx \sum_{\boldsymbol{g}\neq 0} \frac{g_z}{\gamma^3} \frac{\zeta_g}{(\boldsymbol{g}\cdot v_0\hat{\mathbf{z}})} \left(\frac{-\mathrm{i}e^{\mathrm{i}(\boldsymbol{g}\cdot v_0\hat{\mathbf{z}})t} e^{\mathrm{i}(\boldsymbol{g}\cdot\boldsymbol{r}_0)} + \mathrm{c.c.}}{2} \right). \tag{S20}$$

These fluctuations results in photon emission via coherent bremsstrahlung (CB).

1.3 Free-electron radiation in crystalline materials

In this subsection, we present analytical expressions for the radiation produced from two combined mechanisms, parametric X-ray radiation (PXR) and coherent bremsstrahlung (CB), from the results obtained in the previous subsections. It is to be noted that we have generalised the conventional electrodynamics theory, as given by Baryshevsky et al. [1], to consider electrons passing through the crystal with relativistic speeds, but in the limit where electron recoil and quantization of electron momenta is insignificant. Also, while evaluating the number of photons emitted with frequency ω along a given direction $\hat{\mathbf{n}}$, our simulation takes into account all the lattice planes which satisfy the conditions to generate a photon along that direction. We achieve this by summing over the PCB amplitudes $A_{\boldsymbol{g}s}(\omega, \hat{\mathbf{n}})$ generated at frequency ω, due to multiple $\boldsymbol{g}$. The number of $\boldsymbol{g}$ considered is increased until the net PCB amplitude converges, starting from the $\boldsymbol{g}$ that is closest to satisfying the equation $k_g^2 - \omega^2/c^2 = 0$. We assume that the radiation from different electrons is uncorrelated with each other. Therefore, the PCB radiation intensity is linearly proportional to the electron beam current. In the limit $\delta_r << v_0 t$, we simplify equation (1) of the main text as

$$\left\langle \frac{\mathrm{d}^2 N}{\mathrm{d}\omega \mathrm{d}\Omega} \right\rangle \approx \frac{1}{N_e} \frac{\alpha\omega}{4\pi^2 c^2} \sum_{i=1}^{N_e} \left| \sum_{\boldsymbol{g}\neq 0} \left(A_{\boldsymbol{g}s}(\omega, \hat{\mathbf{n}}) \right)_i \right|^2, \tag{S21}$$

where

$$A_{\boldsymbol{g}s}(\omega, \hat{\mathbf{n}}) = A_{\mathrm{PXR}} + A_{\mathrm{CB}}, \tag{S22}$$

$$A_{\mathrm{PXR}} = (v_0 \hat{\mathbf{z}} \cdot \boldsymbol{E}_{\boldsymbol{g}s}) Q, \tag{S23}$$

$$A_{\mathrm{CB}} = \left(\frac{\zeta_g}{\gamma} \frac{1}{2} [\{\boldsymbol{g}; \boldsymbol{e}_s\} + \Lambda] \right) Q + \left(\frac{\zeta_g}{\gamma} \frac{1}{2} [\{\boldsymbol{g}; \boldsymbol{e}_s\} - \Lambda] \right) Q' \tag{S24}$$

$$Q = \frac{1}{2\mathcal{P}}(\sin(2\mathcal{P}t_L)) + \mathrm{i}(\cos(2\mathcal{P}t_L) - 1)), \qquad \mathcal{P} = \frac{\omega - v_0\hat{\mathbf{z}}\cdot(\boldsymbol{k}+\boldsymbol{g})}{2}, \tag{S25}$$

$$Q' = \frac{1}{2\mathcal{P}'}(\sin(2\mathcal{P}'t_L)) + \mathrm{i}(\cos(2\mathcal{P}'t_L) - 1)), \qquad \mathcal{P}' = \frac{\omega - v_0\hat{\mathbf{z}}\cdot(\boldsymbol{k}-\boldsymbol{g})}{2}. \tag{S26}$$

Here the notation $\{\boldsymbol{a};\boldsymbol{b}\}$ denotes

$$\{\boldsymbol{a};\boldsymbol{b}\} = a_x b_x + a_y b_y + \frac{a_z b_z}{\gamma^2}, \tag{S27}$$

and Λ denotes

$$\Lambda = \{\boldsymbol{k};\boldsymbol{g}\}\frac{(v_0\hat{\mathbf{z}}\cdot\boldsymbol{e}_s)}{(\boldsymbol{g}\cdot v_0\hat{\mathbf{z}})}. \tag{S28}$$

In the above expressions, N_e is the number of incident electrons, α is the fine-structure constant, $t_L = L/v_0$ is the interaction time of the electron with the crystal, where L is the interaction length. In equation (S22) the total PCB amplitude $A_{\boldsymbol{g}s}(\omega,\hat{\mathbf{n}})$ is written as sum of PXR amplitude (A_{PXR}) and the CB amplitude (A_{CB}). The PCB radiation intensity (equation (S21)) peaks when $\mathcal{P}\to 0$ and $\mathcal{P}'\to 0$, resulting in $|Q|^2\to t_L^2$ and $|Q'|^2\to t_L^2$, which indicates that the peak has L^2 dependence. In our study the PCB radiation peak is dominated by PXR. The photon energy at the peak radiation intensity (when $\mathcal{P}\to 0$) is obtained as

$$E = \hbar c\,\frac{\beta_0\hat{\mathbf{z}}\cdot(\hat{U}\boldsymbol{g}_0)}{1-\beta_0\cos\theta_{\mathrm{obs}}}, \tag{S29}$$

where $\hbar$ is the reduced Planck's constant, $\beta_0 = v_0/c$, $\hat{\mathbf{z}}\cdot(\hat{U}\boldsymbol{g}_0) = (-\sin\phi_{\mathrm{til}}\cos\theta_{\mathrm{til}})\ g_{0x} + (\sin\phi_{\mathrm{til}}\sin\theta_{\mathrm{til}})\ g_{0y} + (\cos\theta_{\mathrm{til}})\ g_{0z}$, where $\hat{U}$ is the unitary matrix and $\boldsymbol{g}_0$ is the reciprocal lattice vector in the unrotated frame. We define θ_{obs} as the angle between the electron beam and the observation direction, ϕ_{til} as the rotation angle of the crystal with respect to the z-axis, θ_{til} as the angle between the incident electron (along the z-direction) and the [001]

zone axis.

1.4 Bandwidth of PCB peak

In this subsection, we determine the effective bandwidth of the radiation peaks measured in our experiments. The radiation spectrum is determined by the function $|Q|^2 = \left|\frac{\sin(\mathcal{P}L/v_0)}{\mathcal{P}}\right|^2$. To obtain $|Q|$ we consider the $\boldsymbol{g}$ corresponding to the lattice plane whose contribution to the photon emission is dominant, since, the contributions from the other planes do not affect the bandwidth significantly. The spectral width between the zeros of the central peak is denoted $\Delta\omega$, whose relative width is obtained as

$$\frac{\Delta E}{E} = \frac{\hbar\Delta\omega}{\hbar\omega} = 4\pi\frac{v_0}{L\omega}, \tag{S30}$$

where ΔE represents the corresponding energy width. The relative full width at half maximum (FWHM) corresponding to equation (S30) is estimated as

$$\frac{\Delta E_{FWHM}}{E} = \frac{\hbar\Delta\omega_{FWHM}}{\hbar\omega} \approx 5.6\frac{v_0}{L\omega}, \tag{S31}$$

which is obtained by fitting the peaks by a Gaussian function. Equation (S31) is consistent with the expression derived in previous works [7, 8]. However, the measured PCB peak is broadened due to the observation angular spread $\Delta\theta_{\mathrm{obs}}$ and the beam divergence $\Delta\theta_{\mathrm{e}}$. We then obtain the bandwidth as

$$\Delta E_\theta \approx \sqrt{\left(\frac{\partial E}{\partial\theta_{\mathrm{obs}}}\Delta\theta_{\mathrm{obs}}\right)^2 + \left(\frac{\partial E}{\partial\theta_{\mathrm{til}}}\Delta\theta_{\mathrm{e}}\right)^2}. \tag{S32}$$

Using equation (S29), we obtain

$$\frac{\partial E}{\partial\theta_{\mathrm{til}}} = \frac{\hbar c\beta_0\hat{\mathbf{z}}\cdot\left(\partial\hat{U}\boldsymbol{g}_0/\partial\theta_{\mathrm{til}}\right)}{1-\beta_0\cos\theta_{\mathrm{obs}}}, \tag{S33}$$

$$\frac{\partial E}{\partial \theta_{\mathrm{obs}}} = -E \frac{\beta_0 \sin \theta_{\mathrm{obs}}}{1 - \beta_0 \cos \theta_{\mathrm{obs}}}, \tag{S34}$$

where $\hat{\mathbf{z}} \cdot \left(\partial \hat{U} \boldsymbol{g}_0 / \partial \theta_{\mathrm{til}}\right) = \left(\sin \phi_{\mathrm{til}} \sin \theta_{\mathrm{til}}\right) \, g_{0x} + \left(\sin \phi_{\mathrm{til}} \cos \theta_{\mathrm{til}}\right) \, g_{0y} - \left(\sin \theta_{\mathrm{til}}\right) \, g_{0z}$. Taking the EDS detector's energy resolution R into consideration, we obtain the total bandwidth of the measured peaks as

$$\Delta E_{\mathrm{tot,ns}} \approx \left[\left(5.6 \frac{\hbar v_0}{L}\right)^2 + \left(\frac{\hbar c \beta_0 \hat{\mathbf{z}} \cdot \left(\partial \hat{U} \boldsymbol{g}_0 / \partial \theta_{\mathrm{til}}\right)}{1 - \beta_0 \cos \theta_{\mathrm{obs}}} \Delta \theta_{\mathrm{e}}\right)^2 + R^2 + E^2 \left(\frac{\beta_0 \sin \theta_{\mathrm{obs}}}{1 - \beta_0 \cos \theta_{\mathrm{obs}}} \Delta \theta_{\mathrm{obs}}\right)^2 \right]^{1/2}. \tag{S35}$$

In our experiments, the shadowing effect affects the measured bandwidth considerably via modifying the θ_{obs} and $\Delta\theta_{\mathrm{obs}}$ (In equation (S35), the subscript "ns" on the left-hand-side stands for "no shadowing", reminding us that it has not taking shadowing effect into account). We modify equation (S35) to take shadowing effect into account in Section 3.

2 Tuning photon energy via vdW structure tilt for relativistic electrons

In this section, we show the advantage of tunability via vdW structure tilt over tunability via electron energy. As can be seen from Fig. S2a, for relativistic electrons, the photon energy tuning range is enhanced by more than 1000 % at high observation angles ($> 80°$) and it becomes challenging to tune the photon energy by varying electron energy at observation angles beyond 20°. This can also be seen from Figs. S2b,c where the photon energy (in eV) contour lines practically run parallel to the electron energy axis. Therefore, in this regime, the feasible way to tune the photon energy in real-time is by varying the vdW structure tilt angle θ_{til}, as indicated by the red arrows in Figs. S2b,c (which also corresponds to the vertical red lines in Figs. S2d,e respectively) at fixed electron kinetic energy 6 MeV. For

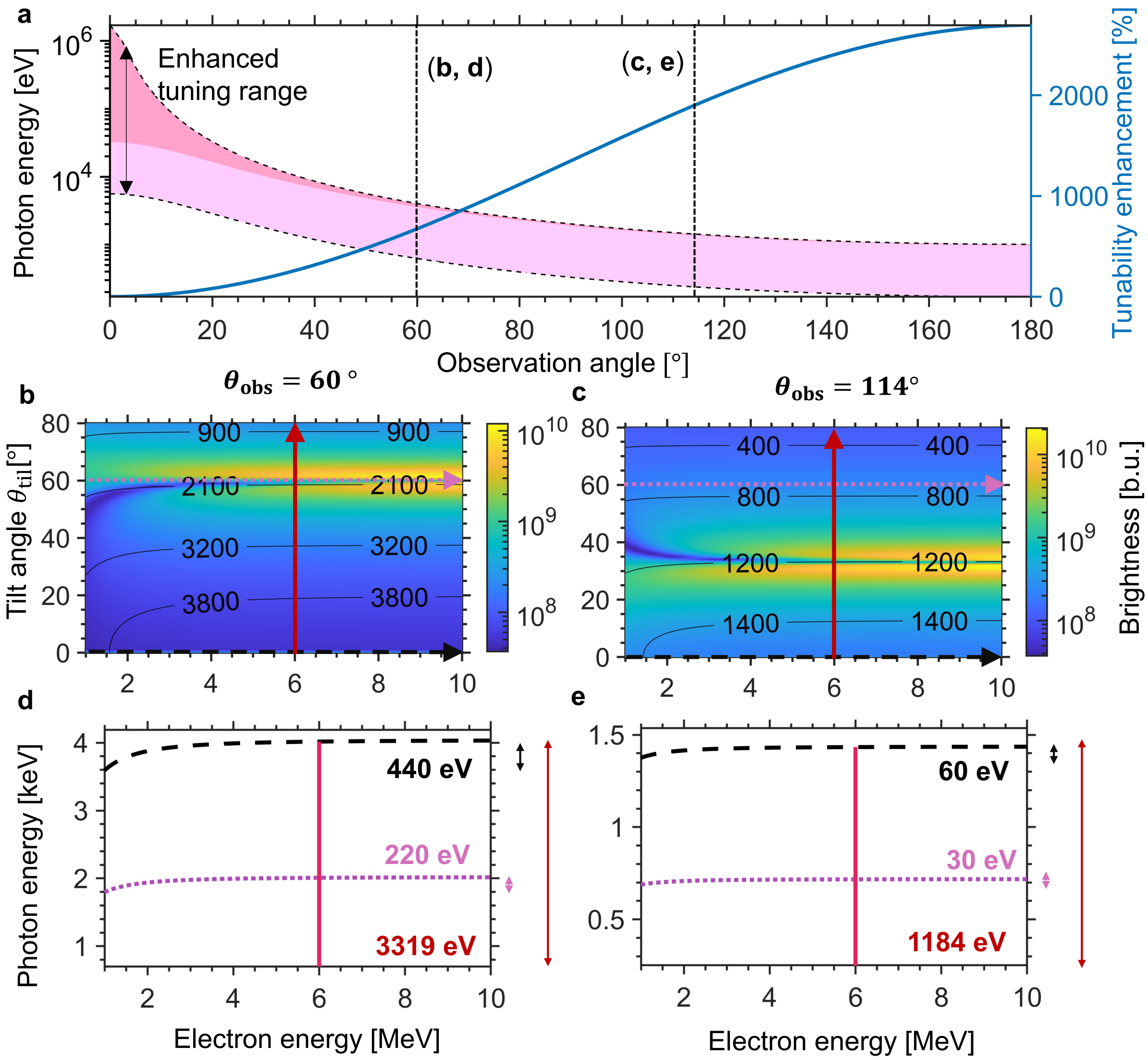

Figure S2: **Enhanced tunability from relativistic electrons for various observation angles in WS_2.** **a** The entire shaded region between the two dashed lines corresponds to the accessible photon energy range when vdW structure tilt and electron energy are simultaneously varied, whereas the darker pink shaded portion corresponds to that when only the electron energy is varied. The blue curve represents the percentage enhancement in the photon energy range in the former scheme. **b**,**c** Brightness (equation (S21)) as a function of electron energy for $\theta_{\rm til}$ from 0° to 80° at different $\theta_{\rm obs}$, where [b.u.] ("brightness units") stands for [photons s^{-1} mrad^2 mm^2 per 0.1% BW]. The photon energy of X-rays (in eV) is indicated by the black contour lines (equation (S29)). **d**,**e** Accessible photon energy range by tuning along the arrows in colormaps (**b**,**c**) respectively. Our enhanced tunability especially favors emission at detector angles beyond 20° and in the soft X-ray range. In this figure, $\phi_{\rm til} = 0°$ and $L = 100$ nm.

$\theta_{\rm obs} = 60°$ (Figs. S2b,d), the accessible photon energy range by varying only electron energy is 440 eV (dashed black line in Fig. S2d). In contrast, the photon energy range is enhanced

by 654 % to 3319 eV, by varying θ_{til} (red line in Fig. S2d). For $\theta_{\text{obs}} = 114°$ (Figs. S2c,e), the accessible photon energy range by varying only the electron energy is 60 eV (dashed black line in Fig. S2e). In contrast, the accessible photon energy range is enhanced by 1873 % to 1184 eV by varying θ_{til} alone (red line in Fig. S2e).

3 Impact of the shadowing effect in X-ray measurements

The observation angle θ_{obs} of the EDS on the JEOL 2010HR TEM is normally 112.5°. The effective observation angle θ'_{obs} is in fact larger by a few degrees due to the shadowing effect, which arises from partial blockage by the sample holder of the emitted X-rays [9]. The shadowing effect is more significant for a small vdW structure tilt angle θ_{til} shown in Figs. S3a,b. On the other hand, the shadowing effect depends on the relative position between the electron incident point and the TEM sample holder edge, shown in Figs. S3a,c,d. By taking shadowing effect into consideration, the effective observation angle is

$$\theta'_{\text{obs}} = \theta_{\text{obs}} - \eta \cos(3\theta_{\text{til}}), \tag{S36}$$

where η describes the relative position between incident point and the sample holder edge. Equation (S36) is only valid for θ_{til} from 0° to 30° (our experimental range on the JEOL 2010 HR TEM). For Fig. 1b of the main text, $\eta = 3$ provides a good agreement between theoretical predictions and experimental results. The corresponding θ'_{obs} is given in Table. S1. The third, fourth and fifth columns of the table display respectively the measured PCB peak position after calibration (E_c) and theoretical predictions without (E_1) and with (E_2) the shadowing effect. Comparing E_c and E_1, we see that the shadowing effect is more significant for a smaller θ_{til}, as expected from the visualization in Fig. S3. For example, $E_1 - E_c$ is 24 eV

and 7 eV for $\theta_{\mathrm{til}} = 0^\circ$ and 25°, respectively. After taking the shadowing effect into account, the theoretical predictions (E_2) agree with the experiment results. E_1 and E_2 are calculated using equation (S29) with the observation angle given by θ_{obs} and θ'_{obs}, respectively.

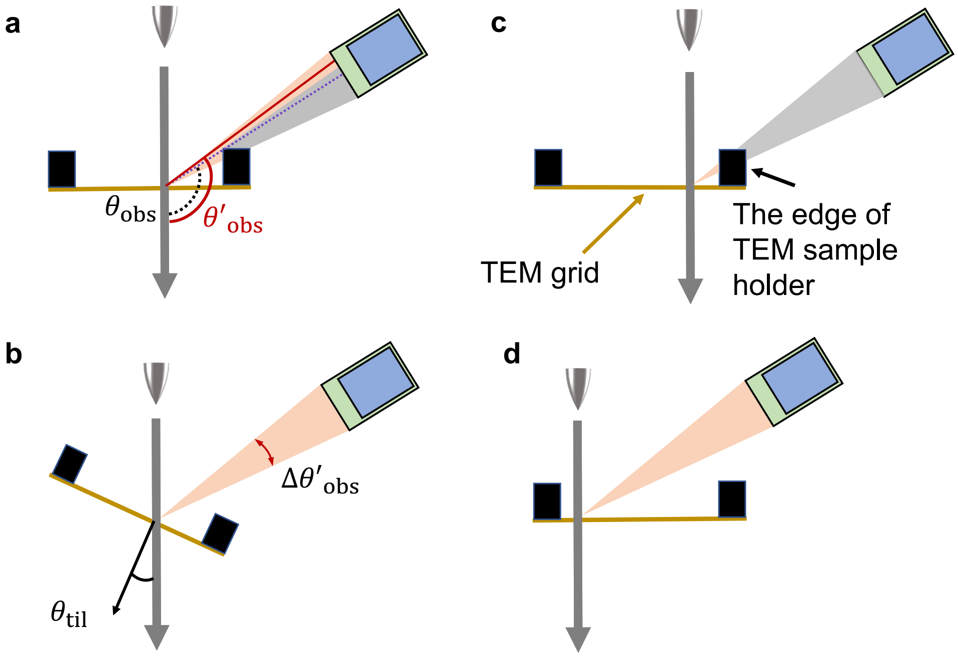

Figure S3: **Shadowing effect arising from partial blockage of the emitted X-rays by the TEM sample holder**. The amount of tilt in the TEM sample holder, and the position of the incident electron beam, affect the effective observation angle and observation angular spread. The effective experimental observation angle and observation angular spread are θ'_{obs} and $\Delta\theta'_{\mathrm{obs}}$ respectively. **a** The shadowing effect results in a larger effective observation angle and a smaller effective observation angular spread. **b** Since the edges of the sample holder are responsible for the shadowing, tilting the sample towards the detector naturally reduces the shadowing effect. The shadowing effect is enhanced when the electron beam is positioned too close to the sample holder edge nearest the detector (**c**); and diminished when the electron beam is positioned farther from the edge (**d**).

As shown in Fig. S3, the shadowing effect affects not only the observation angle but also the observation angular spread $\Delta\theta_{\mathrm{obs}}$. For the JEOL 2010HR TEM, $\Delta\theta_{\mathrm{obs}}$ is approximately 12° without considering the shadowing effect. The influence of the shadowing effect on the observation angular spread is estimated as

$$\Delta\theta'_{\mathrm{obs}} = \Delta\theta_{\mathrm{obs}} - 0.8 \times (\theta'_{\mathrm{obs}} - \theta_{\mathrm{obs}}). \tag{S37}$$

Table S1: Shadowing effect on the output X-rays, where $\eta = 2$ for Fig. 2c (main text) and $\eta = 3$ for the rest of figures. All figures in the table refer to the main text figures.

Fig No.	E_k [keV]	$\theta_{\rm til}[°]$	E_c [eV]	E_1 [eV]	E_2 [eV]	ΔE_c [eV]	ΔE_1 [eV]	ΔE_2 [eV]	$\theta'_{\rm obs}[°]$
1b	200	0	1025 ± 2	1049	1022	128 ± 7	148	125	115.5
1b	200	10	1016 ± 2	1033	1010	134 ± 7	146	127	115.1
1b	200	15	995 ± 2	1013	994	134 ± 7	145	128	114.6
1b	200	20	972 ± 1	985	973	133 ± 6	143	131	114.0
1b	200	25	943 ± 2	950	944	141 ± 8	140	133	113.3
1c	160	0	975 ± 2	992	979	119 ± 6	139	130	114.0
1c	140	0	943 ± 2	956	945	124 ± 6	134	126	114.0
1c	120	0	906 ± 2	915	904	133 ± 8	129	122	114.0
1d	120	30	796 ± 1	792	792	112 ± 7	122	122	112.5
2a	120	0	942 ± 1	968	946	127 ± 4	132	116	115.5
2a	120	10	942 ± 1	953	934	124 ± 4	131	117	115.1
2a	120	20	896 ± 2	909	899	124 ± 6	128	120	114.0
2a	120	30	836 ± 2	838	838	133 ± 5	124	124	112.5
2b	200	0	1070 ± 2	1109	1081	143 ± 5	153	127	115.5
2b	200	10	1066 ± 1	1093	1068	145 ± 4	151	129	115.1
2b	200	20	1023 ± 1	1042	1029	141 ± 3	147	135	114.0
2b	200	30	956 ± 1	961	961	142 ± 3	141	141	112.5
2c	120	0	906 ± 1	921	907	127 ± 7	129	120	114.5
2c	120	10	893 ± 1	907	885	122 ± 5	128	120	114.2
2c	120	20	859 ± 2	866	859	126 ± 9	126	122	113.5
2c	120	25	821 ± 2	835	831	133 ± 6	124	122	113.0
2d	200	0	1019 ± 4	1056	1029	117 ± 14	148	128	115.5
2d	200	10	1016 ± 2	1040	1017	124 ± 7	147	129	115.1
2d	200	20	989 ± 2	992	979	134 ± 8	143	133	114.0
2d	200	30	908 ± 2	914	914	140 ± 7	137	137	112.5

Using equations (S36) and (S37) in place of the observation angle and angular range respectively in equation (S35), we obtain the full width at half maximum (FWHM) of the measured PCB peaks as equation (3) of the main text.

The measured full width at half maximum (ΔE_c) and the theoretical predictions without (ΔE_1) and with (ΔE_2) the shadowing effect are also given in Table S1. A good agreement between ΔE_c and ΔE_2 is observed. Figures 2e,f in the main text are measured by the JEM-ARM300F TEM. The bandwidth of the PCB peaks is approximately equal to the energy resolution of the EDS ,i.e., 75 eV. Under our experimental conditions, the shadowing effect is negligible for $\theta_{\text{til}} > 10°$. Therefore, the experimental and theoretical PCB peaks (Fig. 2e,f of main text) are in good agreement even without taking the shadowing effect into consideration.

4 Energy dispersive X-ray Spectroscopy (EDS) detector calibration

To accurately determine the measured X-ray photon energies, it is necessary to calibrate the energy dispersive X-ray spectroscope (EDS) detector. A well-calibrated EDS detector can determine the photon energy of an X-ray peak within ±2.5 eV of its actual value [10]. To calibrate the EDS detector in our JEOL 2010HR TEM in the low energy region, the Kα peaks of C, O, and Si are measured, as shown in the third column of Table S2. Before calibration, the measured value E_m deviates from the actual value E_i (fifth column) [11] by about 10 eV. The calibration is done using a linear function

$$E_c = 0.9888E_m + 19.5408E_0, \tag{S38}$$

where $E_0 = 1$ eV. The calibrated value of E_c (fourth column) well matches the actual value

Table S2: EDS detector calibration of the JEOL 2010 HR TEM in the low energy region.

Kα	Specimen	E_m [eV]	E_c [eV]	E_i [eV][11]	$E_c - E_i$ [eV]	ΔE_m [eV]
C	Graphite	259.2	275.8	277.0	-1.2	90
O	WO_3	512.5	524.9	526.3	-1.4	94
Si	Si	1739.6	1739.9	1739.7	0.2	100

Table S3: EDS detector calibration of the JEM-ARM300F TEM in the low energy region.

Kα	Specimen	E_m [eV]	E_c [eV]	E_i [eV][11]	$E_c - E_i$ [eV]	ΔE_m [eV]
C	Graphite	259.7	278.5	277.0	1.5	54
N	BN	372.9	390.8	392.4	-1.6	63
Al	Al	1477.4	1486.8	1486.7	0.1	76

within an error of 1.5 eV (second-last column). The corresponding full width at half maximum (FWHM) of the measured peak (ΔE_m) is given in the last column, and can be regarded as the energy resolution R of the EDS detector. By interpolating the data, we determine that R $\approx$ 97 eV in our photon energy range of interest [800 eV, 1400 eV]. Similarly, the EDS detector on the JEM-ARM300F TEM is calibrated by a linear function

$$E_c = 0.9924E_m + 20.7432E_0. \tag{S39}$$

The calibrated values (fourth column) match the actual values within an error of 2 eV, as shown in Table S3. By interpolating the data, we determine that $R \approx 75$ eV for this instrument in our X-ray photon energy range of interest.

Bibliography

[1] V. G. Baryshevsky, I. D. Feranchuk, A. P. Ulyanenkov, *Parametric X-Ray Radiation In Crystals: Theory, Experiment And Applications*, Springer, Berlin, German, **2005**.

[2] J. D. Jackson, *Classical Electrodynamics*, Wiley, ed. 2, **1975**.

[3] G. B. Arfken, H. J. Weber, F. E. Harris, *Mathematical Methods For Physicists* (Academic Press, Waltham, MA, ed. 7, 2013).

[4] Y. Shvyd'Ko, *X-Ray Optics: High Energy Resolution Applications*, Springer, Berlin, German, **2013**.

[5] H. Nitta, *Phys. Lett. A* **1991**, 158, 270-274.

[6] K, Y. Amosov, B. N. Kalinin, A. P. Potylitsin, V. P. Sarychev, S. R. Uglov, V. A. Verzilov, S. A. Vorobiev, I. Endo, T. Kobayashi, *Phys. Rev. E.* **1993**, 47, 2207.

[7] I. D. Feranchuk, A. Ulyanenkov, J. Harada, J. C. H. Spence, *Phys. Rev. E* **2000**, 62, 4225.

[8] K.-H. Brenzinger, B. Limburg, H. Backe, S. Dambach, H. Euteneuer, F. Hagenbuck, C. Herberg, K. H. Kaiser, O. Kettig, G. Kube, W. Lauth, H. Schöpe, T Walcher, *Phys. Rev. Lett.* **1997**, 79, 2462-2465.

[9] R. Pantel, *Ultramicroscopy* **2011**, 111, 1607-1618.

[10] J. I. Goldstein, D. E. Newbury, J. R. Michael, N. W. Ritchie, J. H. J. Scott, D. C. Joy, *Scanning Electron Microscopy And X-Ray Microanalysis*, Springer, New York, USA, ed. 4, **2018**.

[11] G. P. Williams, *X-ray Data Booklet* **2001**.
